# Oxidative Stress and Autophagy Are Important Processes in Post Ripeness and Brown Film Formation in Mycelium of *Lentinula edodes*

**DOI:** 10.3389/fmicb.2022.811673

**Published:** 2022-02-24

**Authors:** Lihua Tang, Ting Chu, Junjun Shang, Ruiheng Yang, Chunyan Song, Dapeng Bao, Qi Tan, Huahua Jian

**Affiliations:** ^1^Institute of Edible Fungi, Shanghai Academy of Agricultural Sciences, Key Laboratory of Edible Fungi Resources and Utilization (South), Ministry of Agriculture and Rural Affairs (China), National Engineering Research Center of Edible Fungi, Shanghai, China; ^2^School of Food Sciences and Technology, Shanghai Ocean University, Shanghai, China; ^3^State Key Laboratory of Microbial Metabolism, School of Life Sciences and Biotechnology, Shanghai Jiao Tong University, Shanghai, China

**Keywords:** *Lentinula edodes*, shiitake mushroom, mushroom development, iTRAQ, metabolomics, oxidative stress, autophagy, brown film formation

## Abstract

*Lentinula edodes* (Berk.) Pegler, the shiitake mushroom, is one of the most important mushrooms in the global mushroom industry. Although mycelium post ripeness and brown film (BF) formation are crucial for fruiting body initiation, the underlying molecular mechanisms of BF formation are largely unknown. In this study, proteomic quantification (relative and absolute) and metabolomic profiling of *L. edodes* were performed using isobaric tags and gas chromatography-mass spectroscopy, respectively. A total of 2,474 proteins were identified, which included 239 differentially expressed proteins. Notably, several proteins associated with autophagy were upregulated, including RPD3, TOR1, VAC8, VPS1, and VPS27. Transmission electron microscopy also indicated that autophagy occurred in post ripeness and BF formation. In time-dependent analysis of the metabolome, metabolites associated with oxidative stress and autophagy changed significantly, including mannitol, trehalose, myo-inositol, glucose, leucine, valine, glutamine, and 4-aminobutyric acid. Thus, oxidative stress and autophagy were important processes in post ripeness and BF formation in *L. edodes*, and new insights were gained into molecular mechanisms at proteome and metabolome levels.

## Introduction

*Lentinula edodes* (Berk.) Pegler, the shiitake mushroom, is one of the most important cultivated mushrooms worldwide (Shim et al., 2016). Shiitake mushrooms have been used for centuries in cooking and as medicine, and they contain many nutritional and bioactive compounds, such as lentinan, an important antitumor agent (Pandya et al., 2019; Mahmood et al., 2021). *Lentinula edodes* was first cultivated at least 800 years ago in China (Tang et al., 2013). The cultivation cycle is very long and consists of four distinct stages: vegetative mycelia growth and colonization, mycelia post ripeness and brown film (BF) formation, primordium initiation, and fruiting body development (Tang et al., 2020). Mycelia post ripeness and BF formation require at least 3 months, are necessary for primordial initiation, and ultimately affect mushroom yield and quality (Tang et al., 2016). Initiation and progression of this second stage are affected by genetic and environmental factors, such as light (Tang et al., 2013; Yoo et al., 2019; Song et al., 2020). Mechanisms of mycelium post ripeness and BF formation and initiation of fruiting bodies in shiitake mushrooms are unknown, especially those involving proteins and metabolism, which have not been reported. The shiitake genome has been fully sequenced (Chen et al., 2016; Sakamoto et al., 2017), and therefore, the complicated biological mechanisms of the second stage can be analyzed at the omics level.

Reactive oxygen species (ROS) are essential signaling molecules in most organisms in response to stress (Waszczak et al., 2018). For example, stress-related genes are activated by light in *Aspergillus nidulans* (Elramli et al., 2019), and ROS affect photomorphogenesis in *Neurospora crassa* (Belozerskaya et al., 2012). Autophagy, from the Greek words “auto” (self) and “phagy” (to eat), is a highly regulated cellular degradation and recycling process that is conserved from yeast to higher eukaryotes (Sibirny, 2011). Autophagy is also important in growth, development, and pathogenesis of fungi (Yang and Klionsky, 2010; Wang et al., 2019). In this study, mechanisms of mycelium post ripeness and BF formation were investigated using isobaric tags for relative and absolute quantitation (iTRAQ)-based proteomics, gas chromatography-mass spectrometry (GC-MS)-based metabolomics, and transmission electron microscopy to analyze cell structures. The results show that autophagy is critical in the second stage and that the process has a special association with activation of age-dependent ROS and oxidative stress. Thus, the study provides new insights for further studies on autophagy-mediated mushroom development and metabolism.

## Materials and Methods

### Fungal Mycelium Growth Conditions and Sampling

Mycelia growth and isolation were performed according to a previously described protocol (Tang et al., 2013). Fungal mycelium was grown in the dark for 30 days to allow full colonization of the substrate (sawdust 31%, bran 8%, gypsum powder 1%, and H_2_O 60%) (sample 1). Then, mycelia were exposed to a 12-h light:12-h dark photoperiod, and samples were collected for proteomic and metabolic analyses every 15 days (i.e., sample 2 at 45 days, sample 3 at 60 days, and sample 4 at 75 days). Two independent biological samples were used in proteomic analyses, and eight independent biological samples were used in metabolic analyses. Color was evaluated according to a previously described protocol (Tang et al., 2013).

### Protein Extraction, Digestion, and Labeling With Isobaric Tags for Relative and Absolute Quantitation

Total proteins were extracted from surface mycelium tissue as previously described with some modification (Tang et al., 2016). Samples were broken and crushed, and 1.0 ml of extraction solution [0.7 M sucrose, 0.1 M NaCl, 0.5 M Tris–HCl (pH 7.5), 50 mM Ethylene Diamine Tetraacetie Acid (EDTA), 0.2% dithiothreitol] was added, followed by 1.0 ml of Tris-saturated phenol solution. Solutions were mixed for 30 min at 4°C, and then also at 4°C, samples were centrifuged at 7,100 × *g* for 10 min. The upper layer was collected following centrifugation. A 5× volume of precooled 0.1 M ammonium acetate–methanol solution was added, and solutions were precipitated overnight −20°C. Solutions were then centrifuged at 12,000 × *g* at 4°C for 10 min and precipitates were collected. A 5× volume of precooled methanol was added to clean, and samples were centrifuged at 12,000 × *g* for 10 min and precipitates were collected. Methanol was removed using acetone. Samples were centrifuged at 12,000 × *g* at 4°C for 10 min and precipitates were collected. After drying at room temperature, precipitates in sodium dodecyl sulfate (SDS) lysates were dissolved. Solutions were centrifuged at 12,000 × *g* at room temperature for 10 min, and supernatants were collected. The extraction was repeated, and combined supernatants represented the total protein solution. Bicinchoninic acid reagent (Thermo Fisher Scientific, Shanghai, China) was used to determine protein content (Reichelt et al., 2016). Total protein (100 μg) was extracted from each sample solution and then digested with Trypsin Gold (50 ng/ml; Beijing Hualishi Technology Co., Ltd., Beijing, China) at 37°C for 12 h, as previously described (Li et al., 2018). The iTRAQ labeling was performed using an iTRAQ 8-plex reagent kit (AB Sciex, Framingham, MA, United States). Isobaric tags for relative and absolute quantitation were used to label peptide solutions of mycelium samples 113 (30 days), 114 (30 days), 115 (45 days), 116 (45 days), 117 (60 days), 118 (60 days), 119 (75 days), and 121 (75 days).

### Peptide Fractionation and Quantitative Proteomic Analysis by Liquid Chromatography-Mass Spectrometry/Mass Spectrometry

Peptides were separated on an Agilent 1200 HPLC with an Agilent column (Wilmington, DE, United States). All analyses were performed on a Triple TOF 5600 System (AB Sciex) fitted with a NanoSpray III source (AB Sciex). Samples were loaded by a capillary C18 trap column and then separated by a C18 column on an Eksigent NanoLC-1D plus system (AB Sciex) using an autosampler with a flow rate of 300 nl/min. Ion spray voltage was 2.5 kV, MS scans were acquired in 250 ms, and product ion scans that exceeded a threshold of 150 counts per second with a two to five charge state were collected at most 35 times. Cycle time was fixed to 2.5 s. Specific parameters were as previously described (Li et al., 2018).

### Database Search and Quantitative Proteome Analysis

The iTRAQ data were processed with Protein Pilot Software v5.0 (AB Sciex) against the *L. edodes* genome database (Chen et al., 2016) using the Paragon algorithm. Experimental data from tandem mass spectrometry were matched with genome data to identify proteins. Reliable proteins were screened using the following parameters: peptide ≥2 and false discovery rate >1%. Proteins with a fold change of >1.5 or <0.67 compared with control levels were considered differentially expressed proteins (DEPs), and results of *t*-tests with *P* < 0.05 indicated significant changes in proteins.

### Gene Ontology Annotation and Bioinformatics Analysis of Differentially Expressed Proteins

For functional classification of DEPs, the multiomics data analysis tool OmicsBean^1^ was used, which integrates Gene Ontology (GO) enrichment and Kyoto Encyclopedia of Genes and Genomes (KEGG)^2^ pathway analyses. A Venn diagram online tool^3^ was used to identify DEPs shared in three compared pairs (2-1: 45 days vs. 30 days; 3-1: 60 days vs. 30 days; and 4-1: 75 days vs. 30 days). When *P* ≤ 0.05, the GO term or KEGG pathway was regarded as significantly enriched in DEPs.

### Verification of Isobaric Tags for Relative and Absolute Quantitation Data by Western Blot and Reverse-Transcription Quantitative Polymerase Chain Reaction

Western blotting was performed to validate protein abundance in the four samples (30, 45, 60, and 75 days). Polyclonal antibodies were prepared by Wuhan Bioyeargene Biotechnology Inc. (Wuhan, Hubei, China). Target sites for the proteins RPD3 and VMA3 were GGNWKMNGDKNQINC and HDSPAVSFCHGRDSPLKLRQ, respectively. Primary antibodies were obtained from New Zealand rabbits. Membranes were incubated for 1 h with horseradish peroxidase (HRP)-conjugated anti-rabbit IgG antibody (1:2,500; ab6721, Abcam, Shanghai, China) as the secondary antibody. Mycelia samples were ground to powder in liquid nitrogen. One hundred microliters of lysate containing 1% Triton X-100, 1% sodium deoxycholate, and 0.1% sodium dodecyl sulfate (Shanghai Biyuntian Biotechnology Co., Ltd., Shanghai, China) was added to 10 mg of tissue in order to crack and degrade the mycelium. The resulting solution was centrifuged at 15,000 × *g* at 4°C for 10 min. A loaded sample was 10 μl, and the reference protein used to calibrate the quantity of loaded samples was β-actin. To validate expression patterns indicated by transcript abundance, four candidate genes related to autophagy were further analyzed by reverse-transcription quantitative PCR (RT-qPCR). Total RNA from each sample was isolated using TRIzol reagent (Invitrogen, Carlsbad, CA, United States) according to the manufacturer’s instructions. The RT-qPCR analysis was conducted on a 7500 real-time PCR system (Applied Biosystems, Foster City, CA, United States). Reverse-transcription qPCR amplification and analysis were performed as described in Tang et al. (2013).

### Metabolite Extraction and Gas Chromatography-Mass Spectrometry

Metabolite extraction for GC-MS was performed according to Zhang et al. (2018). To evaluate the reproducibility of GC-MS during the analysis, a quality control (QC) sample was prepared by mixing aliquots of all samples (a pooled sample). The derivative samples were analyzed on an Agilent 7890B gas chromatography system coupled to an Agilent 5977A mass-selective detector (Agilent Technologies Inc., Santa Clara, CA, United States), which was equipped with an HP-5 MS capillary column (30 m × 0.25 mm × 0.25 μm; Agilent J&W Scientific, Folsom, CA, United States, and Agilent Technologies Inc.). Helium (>99.999%) was used as the carrier gas at a constant flow rate of 1.0 ml/min. Injection volume was 1 μl, and injector temperature was 280°C in split mode. Ion source and interface temperatures were set to 230 and 250°C, respectively. Mass spectra were taken at 70 eV; mass data were acquired in full-scan mode (m/z 35–780); and solvent delay time was set to 8.5 min.

### Gas Chromatography-Mass Spectrometry Data Processing and Statistical Analysis

To assess biological variance, eight biological replicates were extracted and analyzed in parallel under identical conditions. The GC-MS data were processed by XCMS^4^ running under the R package^5^. Variables with <30% relative standard deviation of the QC were retained for further multivariate data analysis. Unsupervised principal components analysis (PCA) and supervised partial least squares discriminant analysis (PLS-DA) (Barbosa et al., 2020) clustering methods were run on GC-MS data in Simca-P software v14.0 (Umetrics AB, Umeå, Sweden)^6^. Unit variance scaling was used in PCA and orthogonal projections latent structures discriminant analysis (OPLS-DA). Quality of models was described by R^2^X or R^2^Y and *Q*^2^ values. Variable importance of projection (VIP) was the weighted sum of squares of the PLS-DA and indicated the importance of a variable to an entire model; metabolites with VIP > 1.5 and *P* < 0.05 were selected. To identify metabolites, the commercial database Fiehn was searched (Mastrangelo et al., 2015). Peaks with a similarity index greater than 80% were tentatively identified as metabolites. Identified metabolites were mapped to general biochemical pathways according to their annotation in the KEGG database^7^. A heat map was generated with R software.

### Transmission Electron Microscopy

Ultrastructure of mycelium samples was investigated *via* transmission electron microscopy (TEM). Surface mycelium samples were removed, fixed in 2.5% paraformaldehyde, and sent to the East China Normal University Electron Microscope Center (Shanghai, China) for sample embedding and slicing and TEM. Thin sections of 70 nm were cut by an ultramicrotome (Leica EM UC7, Wetzlar, Germany), stained with uranyl acetate and lead citrate, and examined and photographed under a JEM-2100 transmission electron microscope (JEOL, Tokyo, Japan). Autophagosome-like structures in TEM sections were quantitated.

## Results

### Brown Film Formation

**FIGURE 1:**
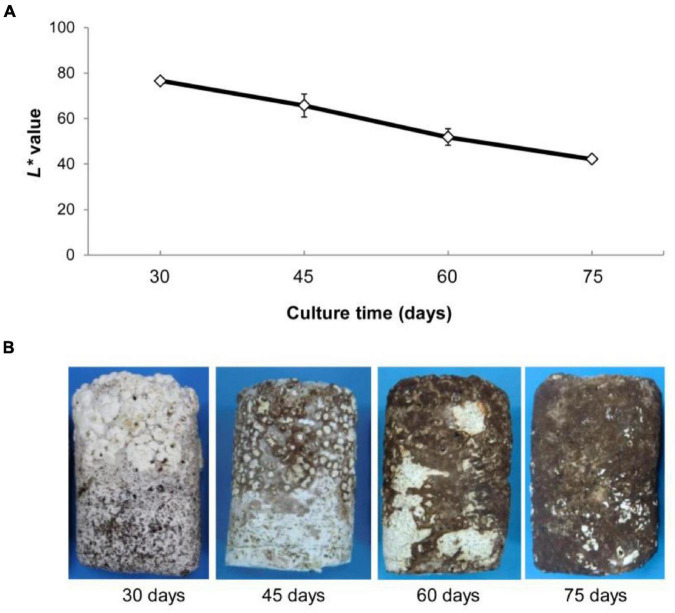
**(A)** Changes in L* values (mean ± SE, *n* = 5) of surface mycelia of *Lentinula edodes* grown under illumination (75 days under a 12-h dark/12-h light regime). L* values represent the lightness of color in the Commission International Eclairage L*a*b* (CIELAB), with maximum L* = 100 representing a perfect reflecting diffuser and minimum L* = 0 representing black. **(B)** Characterization of brown film formation of mycelium at development four times.

Morphological characteristics of mycelium were examined during post ripeness and BF formation at four different times (30, 45, 60, and 75 days after mycelium over growth with pockets). Mycelium gradually developed a BF, which was accompanied by a gradual decrease in the International Commission on Illumination L* (lightness) value from 45 to 75 days (Figure 1A). As shown in Figure 1B, mycelium was white at 30 days without light and then began post ripeness and BF formation stages. Most of the mycelium had BF at 60 days, and the color conversion was complete by day 75 under light irradiation. Thus, shiitake mycelia require a relatively long period to complete post ripeness and BF formation stages.

### Proteomic Expression Patterns in Mycelium During Brown Film Formation

**FIGURE 2:**
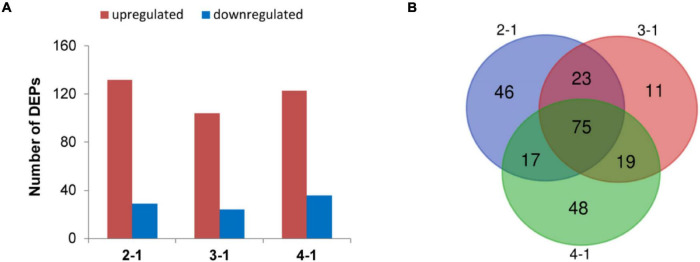
Differentially expressed proteins (DEPs) involved in brown film formation in *Lentinula edodes*. **(A)** Numbers of DEPs that increased (red) and decreased (blue) in accumulation. Comparisons: 2-1, 45 days vs. 30 days; 3-1, 60 days vs. 30 days; and 4-1, 75 days vs. 30 days. **(B)** Venn diagram of DEPs in the three experimental groups.

To investigate the time course of proteome changes, numbers of upregulated and downregulated proteins were determined at the four different times. An iTRAQ-based shotgun quantification approach was used to obtain an overall view of proteomic changes associated with post ripeness and BF formation. In samples of the four stages, a total of 150,867 mass spectra were generated. A total of 2,474 proteins were identified as trusted proteins with unused ≥1.3 and peptides ≥2 (Supplementary Table 1). With a threshold fold-change cutoff of 2.0-fold for increased accumulation and <0.5-fold for decreased accumulation, there were 161 DEP_*S*_ in the 2-1 group, 128 in the 3-1 group, 159 in the 4-1 group (Figure 2A, Supplementary Figure 1, and Supplementary Tables 2A–C). As shown in the Venn diagram (Figure 2B), 75 DEPs were common among the three groups (2-1, 3-1, and 4-1). Groups 2-1 and 3-1 shared 98 DEPs, groups 2-1 and 4-1 shared 92, and groups 3-1 and 4-1 shared 94. Correlations between different proteins in the two samples at each time were relatively good (Supplementary Figure 2). Thus, there were many DEPs, and some key DEPs were in common when samples with BF (45, 60, and 75 days) were compared with those without BF (30 days).

### Functional Annotation and Categories of Differentially Expressed Proteins

**FIGURE 3:**
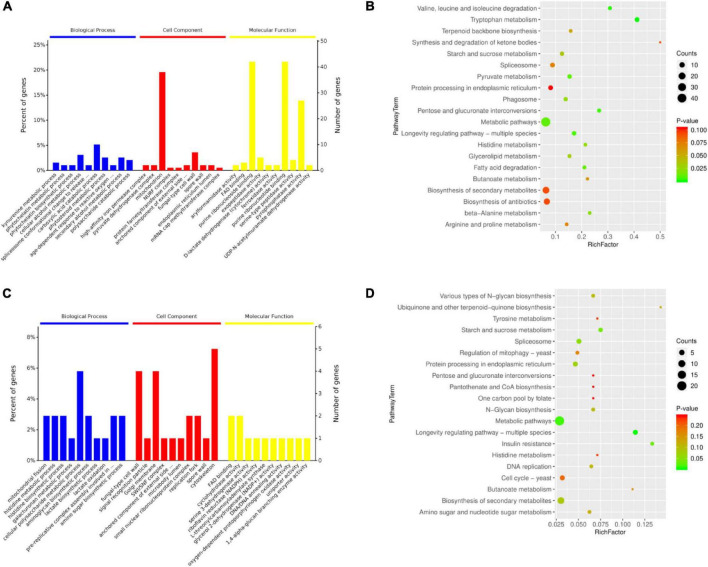
Bioinformatics analysis of **(A,B)** the total 239 and **(C,D)** a subset of 75 differentially expressed proteins (DEPs) in brown film formation in *Lentinula edodes*. **(A,C)** Ten most significantly enriched terms in each category (biological process, cell component, molecular function) in the level 6 gene ontology hierarchy, with the percentage (left) and number (right) of genes involved with a term. **(B,D)** Enriched KEGG pathways clustered into metabolism subcategories, with number of proteins involved in a specific pathway and value of Rich Factor indicated.

The DEPs of the three experimental groups were combined (239 DEPs) (Supplementary Table 3) and analyzed using bioinformatics to identify relevant pathways. In the category biological process (BP), 188 proteins were annotated, and 153 GO terms were enriched. In the category cell component (CC), 192 proteins were annotated, and 27 GO terms were enriched. In the category molecular function (MF), 191 proteins were annotated, and 139 GO terms were enriched. The top ten significantly enriched terms in the GO hierarchy (at level 6) in each category are shown in Figure 3A and Supplementary Tables 4A–C. In BP, kynurenine metabolic process was the most representative term (*P* = 8e-04), followed by a series of metabolic processes and age-dependent responses to ROS, vacuole inheritance, lipid oxidation, maintenance of stationary phase in response to starvation, nucleus-vacuole junction assembly, peroxisome fission, and mitochondrial fission. Thus, several major BP terms were associated with oxidative stress responses and autophagy. In CC, 15.9% of DEPs were in the mitochondrion category (*P* = 0.0389). In MF, catalytic activity was dominant in GO assignments. In KEGG analysis, most metabolic pathways were significantly enriched, including longevity regulating pathway-multiple species (Figure 3B and Supplementary Table 5). Notably, phagosomes were also significantly enriched at *P* = 2.4e-02. To narrow the targets and increase their specificity, the focus was then on the core 75 DEPs that were selected based on GO (at level 6) and KEGG analyses and expression levels (Supplementary Tables 6A–D). As shown in Figure 3C, mitochondrial fission, membrane fission, and age-dependent responses to ROS were also significantly enriched (Supplementary Table 6A). KEGG analysis revealed that longevity regulating pathway-multiple species was also the most representative pathway (*P* = 3.38e-03; Figure 3D). These results indicate that changes in mitochondrial morphology, aging, and ROS are important in physiological processes during BF formation.

### Verification of Isobaric Tags for Relative and Absolute Quantitation Data by Western Blot and Reverse-Transcription Quantitative PCR

**FIGURE 4:**
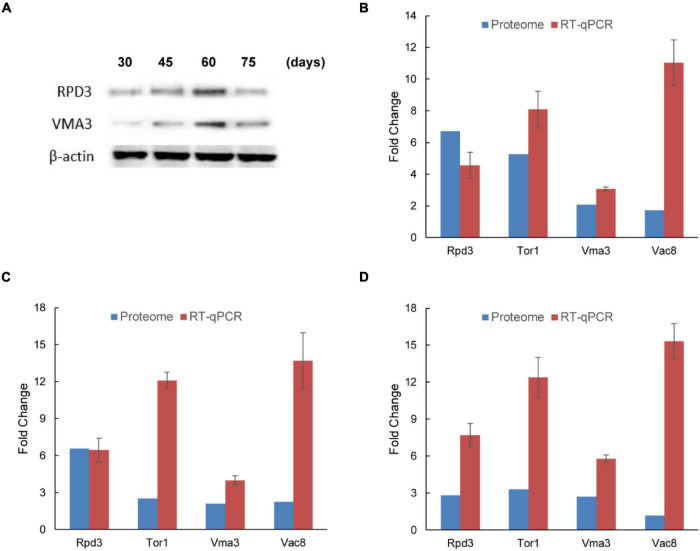
Verification of accumulation patterns of iTRAQ identified proteins in brown film formation in *Lentinula edodes*. **(A)** Western blot analysis of two candidate proteins (RPD3 and VMA3) identified by iTRAQ. Lane 1, 30 days; lane 2, 45 days; lane 3, 60 days; and lane 4, 75 days. **(B–D)** Reverse-transcription qPCR analysis of the transcription levels of four differentially expressed proteins (RPD3, TOR1, VMA3, and VAC8). Comparisons: **(B)** 2-1, 45 days vs. 30 days; **(C)** 3-1, 60 days vs. 30 days; and **(D)** 4-1, 75 days vs. 30 days. Data represent the mean ± SD (*n* = 3).

To verify the changes in protein accumulation measured by iTRAQ analysis, western blot analysis was performed with two selected proteins. As shown in Figure 4A, the accumulation of two proteins (RPD3 and VMA3) from iTRAQ analysis was consistent with the western blot data at each time. The iTRAQ data were also verified at the transcription level with RT-qPCR (Supplementary Table 7). Relative expression of RPD3, TOR1, VMA3, and VAC8 at gene levels was similar to protein level, which also upregulated in the mycelium with BF (Figures 4B–D). VAC8 was highly upregulated at the gene level in 2-1, 3-1, and 4-1 groups. These results indicate that these upregulated expression proteins should be important proteins in BF formation in *L. edodes*.

### Metabolomics of Brown Film Formation

**FIGURE 5:**
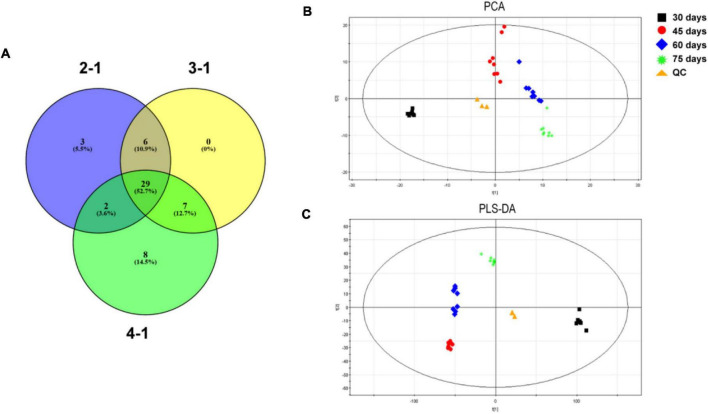
**(A)** Venn diagram of significantly changed metabolites in four experimental groups in brown film (BF) formation in *Lentinula edodes*. Comparisons: 2-1, 45 days vs. 30 days; 3-1, 60 days vs. 30 days; and 4-1, 75 days vs. 30 days. **(B)** Principal component analysis (PCA) and **(C)** partial least squares discriminant analysis (PLS-DA) score plots of metabolic profiles during BF formation.

Metabolites with VIP > 1.0 and *P* < 0.05 were considered statistically significant. The significantly changed metabolites were also screened by *t*-test, and the results are presented in a Venn diagram (Figure 5A and Supplementary Tables 8A–F). Twenty-nine metabolites were common to 2-1, 3-1, and 4-1 groups. In the comparison of 2-1 and 3-1 groups, 35 metabolites were shared; in the comparison of 2-1 and 4-1 groups, 31 metabolites were shared; and in the comparison of 3-1 and 4-1 groups, 36 metabolites were shared. Principal component analysis was performed on the GC–MS data to provide a general overview of clustering among the three comparison groups. In the PCA score plot (Figure 5B), the main model parameters were three principal components, R^2^X = 0.539, and *Q*^2^ = 0.414. The R^2^X value indicated the model was reliable and suitable to explain metabolic differences among the three groups. The four samples were clearly separated from one another, indicating metabolic differences during post ripeness and BF formation. Furthermore, in the PLS-DA (R^2^X = 0.924, R^2^Y = 0.881, and *Q*^2^ = 0.771), the four samples from different times were also separated (Figure 5C), strongly suggesting variation in metabolic processes among samples. Thus, there were large differences in metabolites that could be detected by GC-MS.

### Time Dependence of Metabolism During Brown Film Formation

**FIGURE 6:**
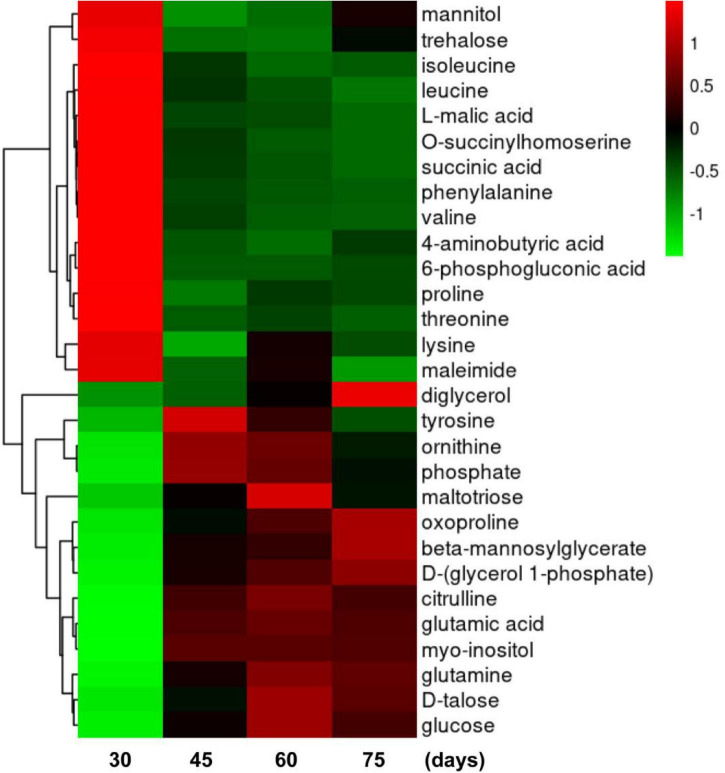
Heat map of clustering analyses of metabolites during brown film formation in *Lentinula edodes.* Color indicates expression level of a metabolite. The higher the expression level is, the brighter the red; the lower the expression level is, the brighter the green. Each horizontal row represents a metabolite through time (days on the *x*-axis).

Further analysis was performed to determine the time-dependent pattern of each metabolite during BF formation. The highest contents of mannitol and trehalose occurred at 30 days, that of tyrosine at 45 days, those of maltotriose and glucose at 60 days, and that of diglycerol at 75 days. Metabolites with similar response patterns were clustered together in a tree in two main groups (Figure 6). Group 1 included mannitol, trehalose, isoleucine, leucine, L-malic acid, O-succinylhomoserine, succinic acid, phenylalanine, valine, 4-aminobutyric acid, 6-phosphogluconic acid, proline, threonine, lysine, and maleimide. Metabolites in group 1 were abundant at 30 days but then decreased from 45 to 75 days. Group 2 primarily included diglycerol, oxoproline, beta-mannosylglycerate, D-(glycerol 1-phosphate), citrulline, glutamic acid, myo-inositol, glutamine, D-talose, and glucose. Levels of group 2 metabolites were greatly elevated from 45 to 75 days, compared with levels at 30 days. In addition, levels of group 2 metabolites tyrosine, ornithine, phosphate, and maltotriose increased initially at 45 days and then decreased dramatically at 75 days. Diglycerol increased from 60 to 75 days and was clearly a group 2 metabolite. Therefore, these metabolites could be used as potential biomarkers of BF formation in *L. edodes*.

### Reactive Oxygen Species and Autophagy During Mycelium Post Ripeness and Brown Film Formation

**FIGURE 7:**
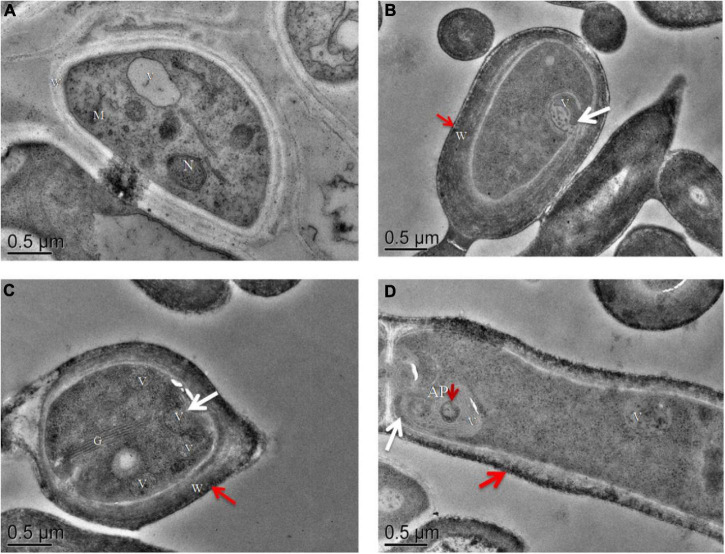
Transmission electron microscopy images of ultrathin sections of cultures of **(A)** mycelia without brown film (BF) (30 days) and **(B–D)** mycelia with BF: **(B)** 45 days, **(C)** 60 days, and **(D)** 75 days. In mycelia with BF, the plasma membrane appears to shrink from the cell wall [**(B–D)**, white arrows]. Autophagosomes (AP) containing recycling components [**(D)**, short red arrow] and a cell wall pigment layer [**(B–D)**, long red arrow] are indicated. N, nucleus; M, mitochondrion; V, vacuole; W, cell wall; G, Golgi. Scale bars = 0.5 μm.

Proteomic and metabolic data suggested that ROS and autophagy were activated during post ripeness and BF formation. Detection of ROS showed that H_2_O_2_ level gradually increased with BF formation, reaching the highest level at 60 days (Supplementary Figure 3 and Supplementary Table 9). In addition, autophagosomal-like structures were observed in TEM micrographs. Nucleus, vacuole, Golgi, mitochondrion, and cell wall were easily distinguished (Figures 7A–D), and characteristic features of autophagy were observed at 45, 60, and 75 days. Vacuolation occurred rapidly, and number of vacuoles increased at 60 days (Figure 7C). The plasma membrane appeared to shrink from the cell wall, and membrane blebbing appeared (Figures 7B–D). At 75 days, an autophagosome (AP) containing recycled components was observed in a larger vacuole (Figure 7D). In addition, the highest average number of vacuoles and autophagosomes contained in each cell was at 60 days (Supplementary Figure 4). Thus, characteristics of autophagy were clearly observed in cells that might be induced by ROS.

## Discussion

### Mycelium Post Ripeness and Brown Film Formation in *Lentinula edodes*

Mycelia were cultured in the dark for vegetative growth. After the substrate was overgrown, mycelia were placed under light to continue cultivation and stimulation. Mycelium then began the post ripeness and BF formation stage. Only a small BF formed at 45 days, and formation was completed at 75 days when the color reached its deepest value. The L* value showed that color always decreased during the post ripeness stage.

### Proteomic Analysis Reveals Crucial Roles of Autophagy Throughout the Post Ripeness Process

Proteomics is a powerful tool that can provide systematic information on the development of fungi and mushrooms. The technique has also been used to investigate autophagy (Wong et al., 2017; Wang et al., 2018). Autophagy is a process of intracellular degradation that is conserved in eukaryotic cells ranging from yeasts to higher plants (Pohl and Dikic, 2019). In fungi, autophagy acts in many critical biological processes, including growth and development and in responses to biotic and abiotic stresses (Zhang et al., 2017; Zheng et al., 2018). To date, there has been little investigation of autophagy-mediated development in edible fungi and mushrooms. In this study, proteomics was used to examine protein profiles during mycelium post ripeness and BF formation and improve understanding of the roles of oxidative stress and autophagy in the development of mushroom mycelia and fruit bodies.

To analyze proteome-level changes during post ripeness and BF formation, mycelium samples with BF were compared with samples without BF. Seventy-five DEPs were annotated, and some were involved in several autophagy-related processes. The protein RPD3 is a class I histone deacetylase that reverses lysine acetylation and regulates autophagy by regulating AP size and frequency of formation and controlling activity through direct deacetylation of autophagy-related proteins (Yi et al., 2012; Cai et al., 2018). In this study, RPD3 was upregulated in mycelia with BF, indicating that posttranscriptional regulation, including acetylation, was involved in BF formation. The vacuolar membrane protein VAC8 is involved in vacuolar membrane dynamics and is also responsible for several autophagic pathways, such as nucleus-vacuole junction formation (Jeong et al., 2017). Vacuolar protein sorting 1 (Vps1) regulates vacuolar membrane fission and fusion and addresses oxidative stress (Arlt et al., 2015). The protein Vps27 is required for TORC1 to specifically modulate micro autophagy (Hatakeyama and De Virgilio, 2019). In this study, differences in expression of VAC8, VPS1, and VPS27 indicated that autophagy-related proteins were activated, suggesting that autophagy-related processes occurred during mycelium post ripeness and BF formation. Further research is required to confirm this speculation.

Nucleus-vacuole junctions are membrane contact sites formed through specific interactions between Vac8p and Nvj1p that mediate a unique autophagy process (Tsai et al., 2014). Oxidative stress is caused by supraphysiological production of ROS, which can cause cellular injury associated with lipid oxidation and autophagy (Lu et al., 2015). Peroxisomes are major sites of ROS production in eukaryotic cells, and peroxisome fission is important in aspersorium-mediated infection of the rice blast fungus, which relies on both glucose-induced and constitutive pexophagy (Mao et al., 2014). Mitochondrial fission has been linked to both mitophagy and global autophagy and contributes to quality control by removing damaged mitochondria during high levels of cellular stress (Youle and van der Bliek, 2012). In this study, the GO terms associated with autophagy were significantly enriched, indicating that autophagy occurred. In addition, the age-dependent response to ROS was enriched among biological processes. This result indicated that ROS signaling induced stress responses in an age-dependent manner and might be important in acclimating to the environmental stress that occurs during post ripeness and BF formation.

### Metabolomics Analysis Reveals Metabolites Involved in Autophagy

To gain insights into changes in metabolic profiles during BF formation, a GC-MS-based metabolomics approach was used. Metabolites are the final products of gene expression, and the metabolome is directly linked to cell physiology. In this study, metabolic flux analysis clearly demonstrated that contents of most oxidative stress- and autophagy-related metabolites changed significantly. Mannitol, which is the most widely recognized polyol in fungi and can quench ROS, decreased with time (Wang et al., 2012). Trehalose, which is important as a free radical scavenger and protects against oxidative stress by regulating autophagy pathways (Mizunoe et al., 2018), also decreased at 30 days. With a decrease in trehalose content, ROS scavenging weakens, which inevitably leads to excessive accumulation of ROS (Zhao et al., 2019). However, myo-inositol and glucose contents increased with BF formation. Myo-inositol is a ubiquitous molecule that has diverse roles in vesicle trafficking, signal transduction, auxin perception, and biotic and abiotic stress responses (Tan et al., 2013). Light can promote inositol biosynthesis to prevent light-induced oxidative stress, which establishes a molecular link between light signals and basal metabolism of inositol (Ma et al., 2016). Glucose metabolism regulates autophagy by controlling cellular ATP, redox status, and signaling. It also regulates autophagy at multiple levels by modulating glycolysis, endoplasmic reticulum stress, and cellular levels of glutathione and ROS (Ma et al., 2013). Thus, decreases in mannitol and trehalose and increases in myo-inositol and glucose may lead to ROS production and autophagy. Notably, among amino acids, leucine, isoleucine, and valine showed the largest decreases, whereas glutamine showed the largest increase. Amino acids are well known regulators of autophagy, and leucine and glutamine are the most important regulators of mTORC1 activity and autophagy (Carroll et al., 2015). In particular, leucine inhibits autophagy at least in part by stimulating mTOR-mediated signaling (Lorin et al., 2013). Glutamine can be converted to α-ketoglutarate, which regulates mTORC1 activity and autophagy (Tan et al., 2017). Thus, these results suggested that metabolites were associated with oxidative stress and autophagy during BF formation, which is consistent with a previous report that found important roles of metabolites in autophagy (Yang et al., 2019).

### Autophagy-Related Ultrastructures in Post Ripeness and Brown Film Formation of *Lentinula edodes*

Autophagy is essential for proper development in higher eukaryotes (Ren et al., 2017). In this study, autophagy emerged at 45 days (Figure 7B) in the post ripeness stage of *L. edodes*. During BF formation, characteristics in some developing mycelium cells indicated autophagy was involved in post ripeness and BF formation. Notably, number of vacuoles increased at 60 days, followed by a sharp decrease at 75 days. These results indicated that vacuole fusion and enlargement might have occurred. Vacuole size was highly dynamic and dependent on growth conditions, and changes in vacuole morphology in response to the environment were also important. Vacuole fusion and morphological changes may be an important link in mushroom development, which are worthy of further study. Some hyphae of plant and human fungal pathogens can grow under severely nutrient-limited conditions by expanding the vacuolar space rather than by synthesizing new cytoplasm and organelles (Veses et al., 2008). Autophagy is a mechanism by which eukaryotes degrade their own unimportant proteins or organelles under starvation or other adverse conditions to help cells survive adversity and is closely associated with growth, development, and reproduction (Zhou et al., 2017). In this study, autophagosome fusion into vacuoles was observed at 75 days, with autophagolysosomes forming to degrade contents. Reactive oxygen species are by-products of aerobic cellular metabolism that are also signal molecules widely involved in regulation of different biological processes, including autophagy. In this study, the highest accumulation of H_2_O_2_ was at 60 days, with concentration then decreasing to normal at 75 days. The pattern with H_2_O_2_ was similar to that observed in changes in number of vacuoles. Reactive oxygen species can activate the vacuole function of processing waste protein in cells (Zhang et al., 2016). However, ROS are also strictly regulated, because at high concentrations, they can be toxic molecules. In that scenario, the level of autophagy is too high, which destroys cell stability and causes apoptosis (Boone et al., 2017). A pigment layer also formed in the cell wall and pigmentation increased in this study, consistent with a previous study that also linked pigmentation and autophagy (Oliveira et al., 2016). Autophagy involvement in the biogenesis of fungal development was recently studied, and a crucial role was demonstrated in development and differentiation of *Botrytis cinerea* (Oliveira et al., 2016) and *Metarhizium robertsii* (Duan et al., 2013). Although autophagy is highlighted in some reports on development and secondary metabolism of fungi, some related characteristics have also been recorded (Khan et al., 2012; Liu et al., 2016). Unfortunately, the role of autophagy in mushroom development remains unclear. Additional studies are needed to understand the general role of autophagy in mushroom development as well as its specific role in mycelium and fruit body development in *L. edodes*.

## Conclusion

*Lentinula edodes* was used as the model system in this study, and proteomics, metabolomics, and TEM were used to characterize changes in ROS accumulation and autophagy during mushroom mycelium development to post ripeness and BF formation. The data in this study indicate that autophagy is an important process in mushroom development. Based on the findings, a potential cascade of cellular events constituting mycelium development and autophagy is proposed (Supplementary Figure 5). Starvation and aging act as signals to induce oxidative stress in mycelia cells, leading to mitochondrial fission, membrane fission, peroxisome fission, and lipid oxidation. Reductions in ROS-quenching substances, including mannitol and trehalose, and increases in ROS-stimulating substances, including myo-inositol and glucose, trigger an ROS burst and promote autophagy by 4-aminobutyric acid. Simultaneously, amino acid metabolism also promotes induction of autophagy (nucleus-vacuole junctions and regulation of mitophagy) by mTOR. In this process, the deacetylase RPD3 may also be involved and eventually promotes nutrient recycling, pigmentation, and primordium initiation through autophagy. Thus, this study improves understanding of autophagy-dependent mechanisms of post ripeness and BF formation and fruit body initiation in *L. edodes*, while also highlighting the critical importance of autophagy in mushroom development in general.

## Data Availability Statement

The mass spectrometry proteomics data have been deposited to the ProteomeXchange Consortium (http://proteomecentral.proteomexchange.org) via the iProX partner repository with the dataset identifier PXD 026391.

## Author Contributions

LT, DB, and QT designed the research. LT, CS, and HJ performed the research. RY and JS analyzed the data. LT and TC wrote the manuscript. All authors contributed to the article and approved the submitted version.

## Conflict of Interest

The authors declare that the research was conducted in the absence of any commercial or financial relationships that could be construed as a potential conflict of interest.

## Publisher’s Note

All claims expressed in this article are solely those of the authors and do not necessarily represent those of their affiliated organizations, or those of the publisher, the editors and the reviewers. Any product that may be evaluated in this article, or claim that may be made by its manufacturer, is not guaranteed or endorsed by the publisher.

## Footnotes

1
http://www.omicsbean.cn

2
https://www.kegg.jp/

3
https://bioinfogp.cnb.csic.es/tools/venny/index.html

4
http://www.bioconductor.org

5
http://www.r-project.org

6
www.umetrics.com/simca

7
http://www.kegg.jp/kegg/pathway.html
