# Supplementary material for: Oxidative Stress and Autophagy Are Important Processes in Post Ripeness and Brown Film Formation in Mycelium of Lentinula edodes
Source: Front Microbiol. 2022 Feb 24;13:811673. doi: 10.3389/fmicb.2022.811673 (PMC8908433; doi:10.3389/fmicb.2022.811673)
Supplement: Supplementary file 1 [file Data_Sheet_1.doc]

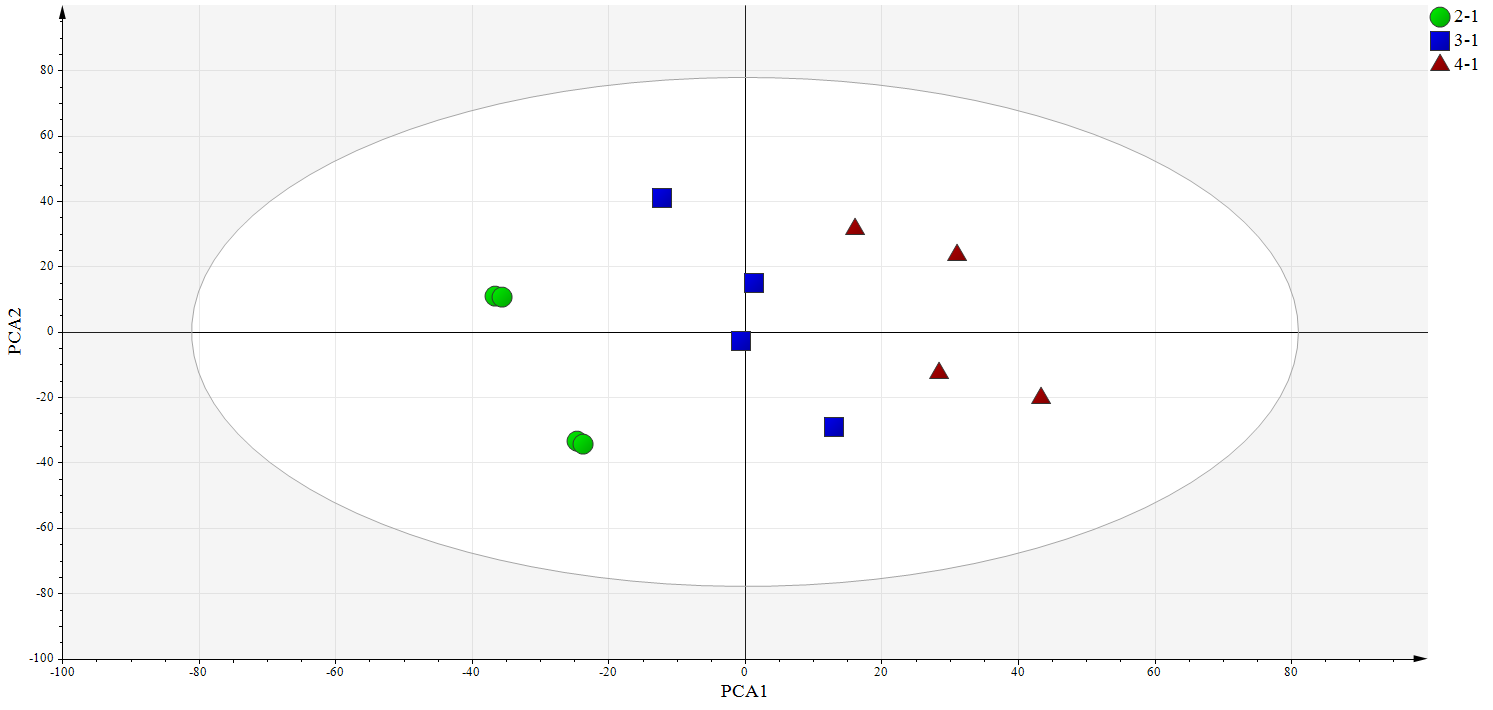
Supplementary Figure 1. The result of PCA (principal component analysis) of the differential proteins (2-1, 3-1, and 4-1 respectively represent the differential proteins between 45 days and 30 days, 60 days and 30 days, and 75 days and 30 days).


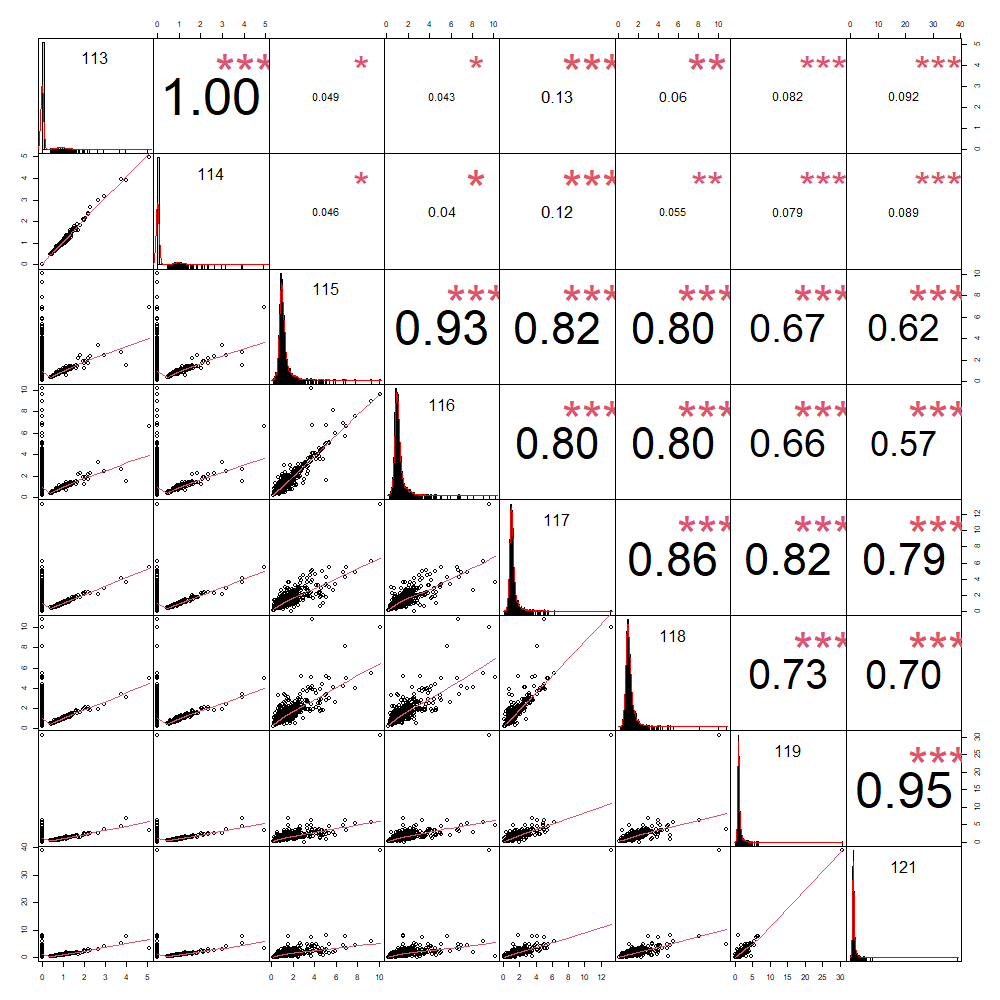


Supplementary Figure 2. The correlation analysis between samples


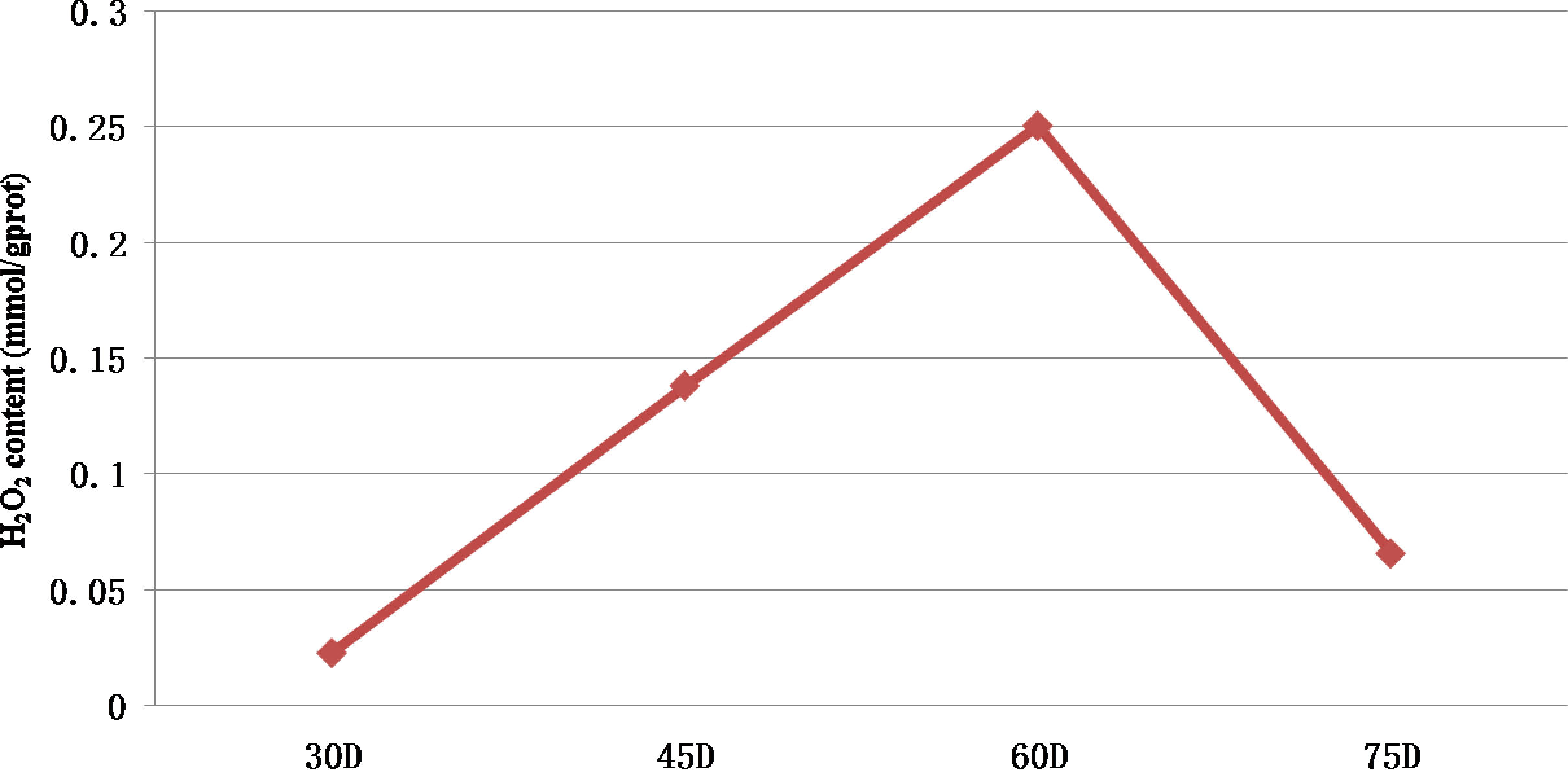


Supplementary Figure 3. The result of ROS detection. The result showed that the H_2_O_2_ level gradually increased during BF formation stage, and reached the highest on the 60th day (30D: 30 days; 45D: 45 days; 60D: 60 days; 75D: 75 days).


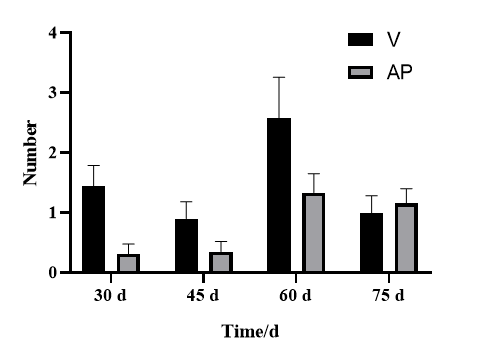


Supplementary Figure 4. The average number of vacuoles and autophagosomes at four-time point during BF formation. V: vacuole; AP: autophagosome.


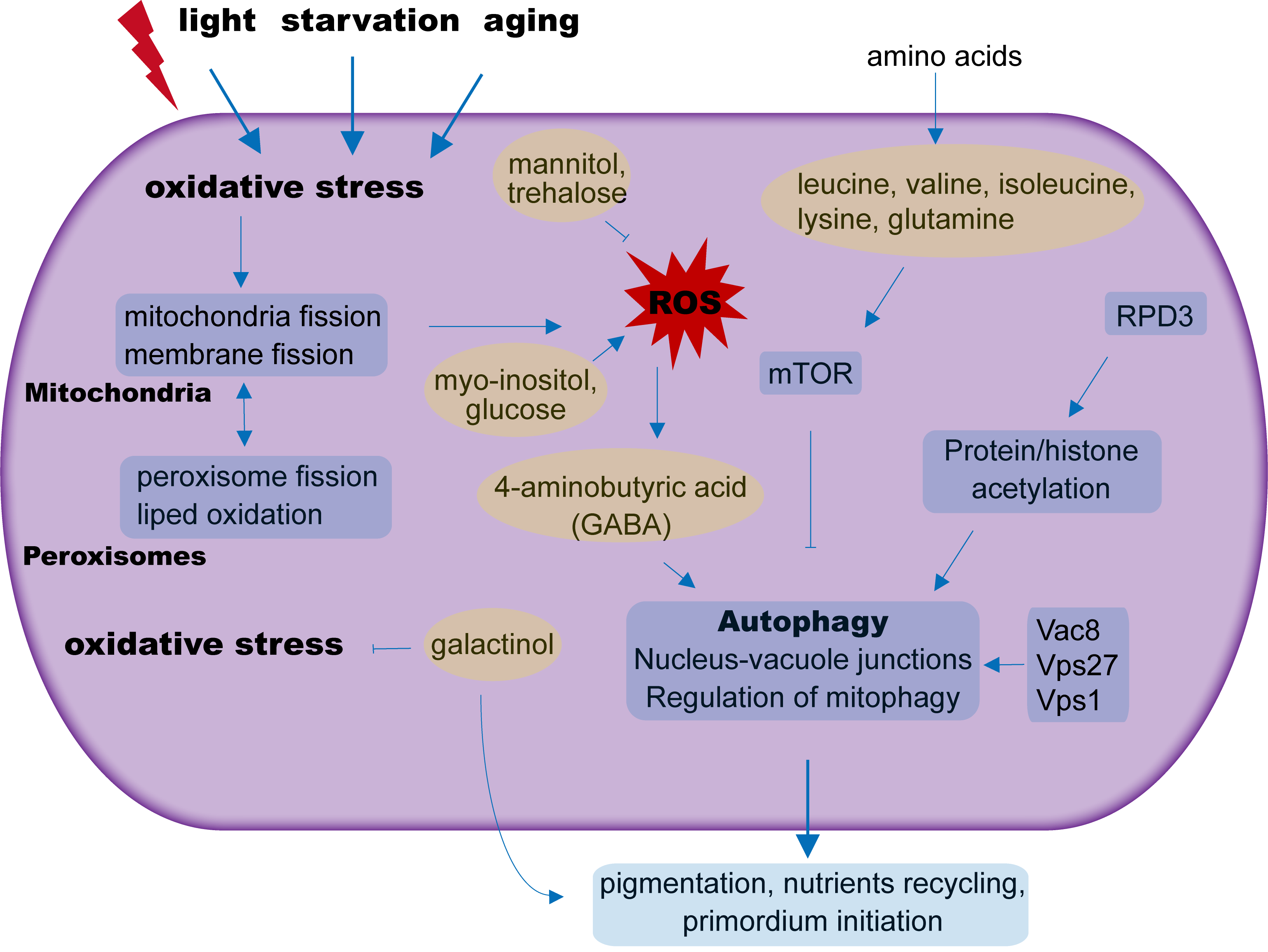


Supplementary Figure 5. Summary: Autophagy is involved in the physiological maturation and BF formation. Starvation, and aging act as signals to induce oxidative stress in mycelial cells, leading to mitochondrial fission, membrane fission, peroxisome fission, lipid oxidation, a reduction in ROS-quenching substances, including mannitol and trehalose, and increases in myo-inositol and glucose, which results in increases in ROS, triggering an ROS burst and promoting autophagy by 4-aminobutyric acid (GABA). At the same time, amino acid metabolism also promotes the induction of autophagy (NVJs and regulation of mitophagy) by mTOR. In this process, deacetylase RPD3 may also be involved in the occurrence of autophagy, eventually promoting nutrient recycling, pigmentation, and primordium initiation through autophagy.
